# Oxytocin measurements in saliva: an analytical perspective

**DOI:** 10.1186/s12917-023-03661-w

**Published:** 2023-07-28

**Authors:** Marina López-Arjona, María Botía, Silvia Martínez-Subiela, José Joaquín Cerón

**Affiliations:** 1 Department of Animal and Food Science, Universitat Autònoma de Barcelona, Bellaterra, Barcelona, 08193 Spain; 2 Interdisciplinary Laboratory of Clinical Analysis, Regional Campus of International Excellence ‘Campus Mare Nostrum’, University of Murcia (Interlab-UMU), University of Murcia, Campus de Espinardo s/n, Murcia, 30100 Spain

**Keywords:** Enzyme immunoassay, Extraction, Oxytocin, Saliva, Welfare

## Abstract

Oxytocin has traditionally been known for its physiological effects on muscle contraction associated with birth and lactation, but in the last years is widely used as a biomarker of “positive experiences” in psychology and behavior. Different types of samples have been used for oxytocin measurements with saliva samples having the particular advantage of an easy and non-stressful collection. However, the low concentration of oxytocin in saliva can represent a limitation for its use. For this reason, sensitive assays and even a previous sample treatment in some cases are required for saliva oxytocin quantification. In addition, the lack of standardized and generally agreed-upon approach to peripheral oxytocin measurement leads to large discrepancies between different laboratories, that use different sample treatment protocols and different assays. The main objectives of this review are to describe the current status of the use of saliva for oxytocin measurement, provide details of the different sample processing techniques that can be applied and inform about the analytical techniques and assays available in different animal species, and also in humans for comparative purposes. It is expected that this information can contribute to an increase in the knowledge about the measurements of oxytocin in saliva and to its wider use in the future.

## Background

### Structure and synthesis

**Fig. 1:**
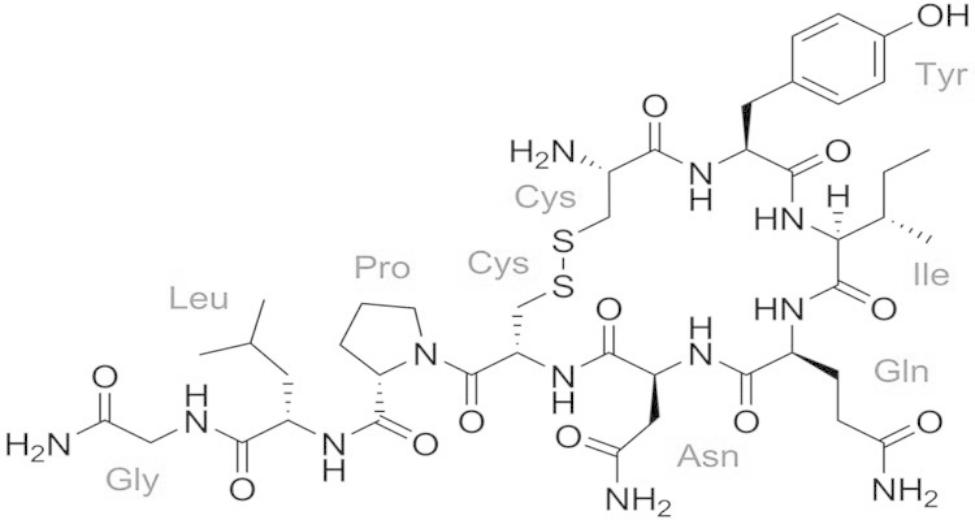
The chemical structure of oxytocin (C_43_H_66_N_12_O_12_S_2_) MW = 1007,19 g/mol [2]

Oxytocin is a hormone composed of nine amino acids (CYIQNCPLG) which in many biological samples can have a cyclic chemical structure by the join of disulfide bonds between cysteine residues 1 and 6, resulting in a cyclic core comprising six amino acids with a flexible three-residue amidated tail [1–3] (Fig. 1). The oxytocin molecule was discovered in 1950 [4] and synthesized for the first time in 1953 [5]. Mammals produce oxytocin, while fish produce isotocin, and amphibians, reptiles and birds have mesotocin, which are oxytocin-like peptides [6]. In addition, oxytocin like peptides are found in a large number of invertebrate species such as mollusks, nematodes, and arthropods with some differences in their structure [7].

Oxytocin is synthesized in the supraoptic and paraventricular nucleus of the hypothalamus[8]. It is transported along with the neurophysins to the posterior lobe of the pituitary gland and then it is released to the blood [8, 9]. From the pituitary gland, oxytocin pathways are distributed in many brain regions associated with the stress response, including the bed nucleus of the stria terminalis and the amygdala [10]. Moreover, oxytocin receptors have been found in some limbic structures, including the bed nucleus of the stria terminalis, central nucleus of the amygdala, septum, and hippocampus [11]. In addition to brain, oxytocin is produced in several peripheral tissues and organs, such as the uterus, ovaries, testis, vascular endothelium, and heart [12].

Oxytocin has some similarities with arginine-vasopressin, another neuropeptide synthesized in the hypothalamus, which shares with oxytocin seven of nine amino acid sequences, differing only in two amino acids [3]. However, several of the known effects of arginine-vasopressin are opposite to those of oxytocin [3] and from the analytical point of view, oxytocin assays should not have cross-reactivity with vasopressin.

### Function and biological relevance

Traditionally, oxytocin is known for its physiological effects on muscle contraction associated with labor (uterine contraction) and lactation (milk ejection) [13]. In addition, some reports stated that oxytocin acts as a stress responsive hormone [14–16]. However, in the last years, oxytocin has been widely studied in the field of psychology and behavior, being considered as a biomarker of positive emotions since it increases in calming and relaxing situations [17]. This hormone plays an important role in social bonding[18] with both short-term and long-term effects[19] and also in birth, lactation, and maternal-filial relationship [20], being in general released during positive interaction situations [21]. Moreover, it improves social memory, social recognition and attention[22] and it also has a strong anxiolytic and stress-reducing effects [23, 24]. Overall, contrarily to most biomarkers used for evaluating welfare, such as cortisol, catecholamines or alpha-amylase that usually are associated with stress and negative situations, the oxytocin has the particularity of being associated with positive experiences. However, while oxytocin was first described as a pro-social hormone, more recent research suggests that oxytocin also increases in non-pro-social behaviors [25]. In addition, oxytocin has an important role in regulation of the immune system[26, 27] and anti-inflammatory effects.

Several mechanisms have been postulated to explain the effects of oxytocin, such as the potentiation of neuronal activity in areas of the brain related to cognitive processes, and the reduction of activity in areas that control the autonomic nervous system [28] as well, the modulation of serotonin activity leading to a reduction of anxiety [29] and interactions with the dopaminergic system and endogenous opioid system [30]. Oxytocin also plays important roles in peripheral tissues, which can feed-back to the central nervous system with routes of connection described [31, 32]. For example, inhaled oxytocin can reach the central nervous system [33], and a route for active transport from blood circulating oxytocin into the brain has recently been discovered in mice [34].

### Analytical issues

There are many studies about peripheral oxytocin measurements that use blood plasma samples [3, 35, 36]. However, saliva has gained a high interest for the measurement of oxytocin in trials related with behavior and stress, since it is an ideal sample due to its non-invasive nature and its easy collection, although the difficulties for assay development and its usually low concentration at these samples can represent a limitation for its use [37].

**Fig. 2:**
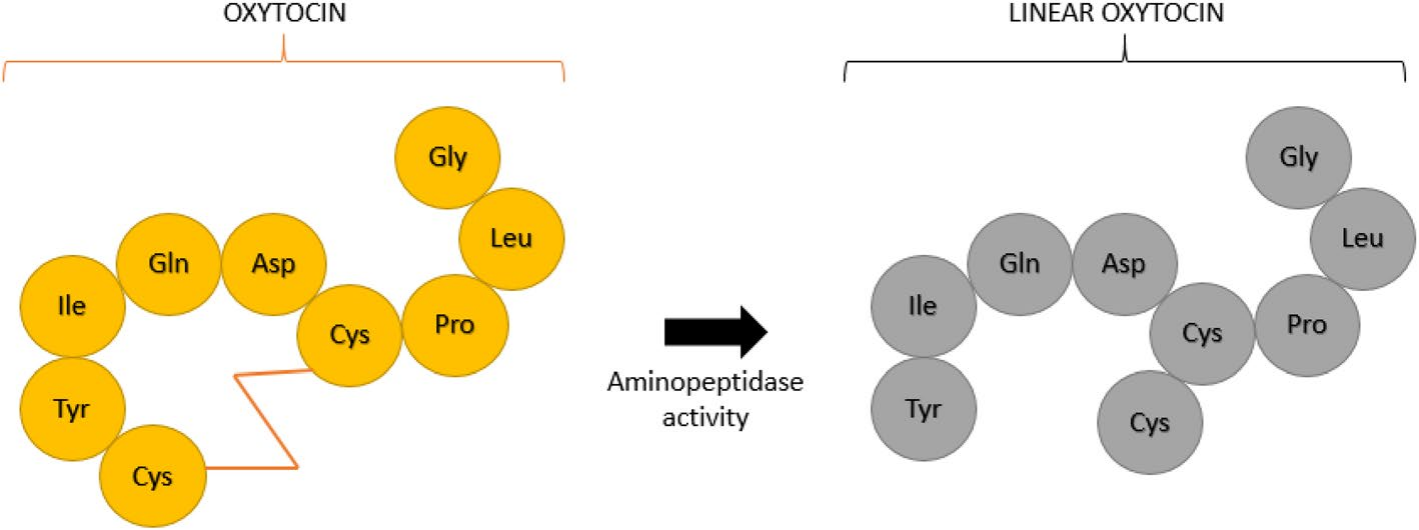
Oxytocin can be degraded by aminopeptidase activity from to its cyclic form (left side from the observer) into a linear form (right side) [33, 38]

In general, there is little consensus on how to manage the saliva samples, the assays that should be used to measure oxytocin and how to interpret the often-discordant results of various methods [38]. In this line, an issue that it is known is a matter of controversy is what the different assays are measuring. Oxytocin can be present in a cyclic or linear form (Fig. 2) that can be differently recognized by the diverse antibodies used in the assays; and also some assays involve sample treatments, such as a reduction-alkylation process that irreversibly breaks disulfide bridges and produces the linear form of oxytocin [39]. These forms can have different psychophysiological effects [37, 40]: the cyclic molecule may act to initiate social interaction, whereas linear oxytocin and C-terminal fragments may induce relaxation and anti-stress effects following social interaction, and anti-inflammatory and antioxidant effects [40]. The linear form can also be formed by an enzymatic degradation[41, 42]. In addition, the oxytocin can bind to proteins but also be in free form, and there can be oxytocin metabolites that can be measured in variable degree by the different assays [38, 43].

### Objectives of this review

The main objectives of this review are to report the current status of the use of saliva for oxytocin measurement, describe the options of sample management and processing and provide an update about the analytical techniques described for the measurements of oxytocin in saliva, in different animal species and also in humans for comparative purposes.

## Use of saliva for oxytocin measurement

Although blood plasma is also frequently used to measure peripheral oxytocin concentrations [3, 35, 36], this review will be focused on saliva samples. The secretion of oxytocin to saliva from central nervous system could be influenced and controlled by autonomic nerves [44] and the circulating molecules in blood plasma are thought to transfer to salivary glands via surrounding capillaries [45], since lipid insoluble molecules such as oxytocin, could enter into saliva mainly via the tight junctions between acinar cells through ultrafiltration [46]. However, more studies should be needed to clearly elucidate the mechanisms and sources of the presence of oxytocin in saliva. In this line, to our knowledge, there are no studies that have evaluated if oxytocin can be synthesized directly in salivary glands.

Saliva has various advantages compared to other sample types that have been used for oxytocin measurements such as cerebrospinal fluid[47] or serum [48–50], such as its easy, non-invasive and quick collection. In addition, saliva sampling usually reduces various sources of stress that are likely to interfere with oxytocin system activity, since it does not require medical care or a laboratory setting.

Table 1 shows different correlations between peripheral (blood and saliva) and central (cerebrospinal fluid) oxytocin concentrations. This table shows a large divergence of correlations between different samples depending on the study. This indicates that, in general, the relationships between oxytocin concentrations in saliva, blood, and cerebrospinal fluid and the possibility of use of peripheral oxytocin quantification to evaluate cerebrospinal fluid concentrations and central oxytocin bioavailability are not yet fully well-understood [33]. Martin et al. [51] found that oxytocin in cerebrospinal fluid showed a high correlation with saliva but a low correlation with plasma, so saliva oxytocin could help to assess cerebrospinal oxytocin levels in patients in neurocritical care. In this line, other authors have also reported that plasma oxytocin levels do not predict central concentrations [51, 52].

In addition, there is in general no high correlation between plasma and saliva concentrations. Quintana et al.[33] concluded that salivary oxytocin concentrations do not accurately reflects plasma oxytocin concentrations after intranasal or intravenous oxytocin administration in men, and Javor et al.[53] did not found correlation between salivary and plasma oxytocin concentrations in healthy young men. Also, according to Martins et al. [54], salivary oxytocin is a weak surrogate for plasmatic oxytocin in humans (r = 0.10). However, also here, there is some degree of divergence between studies since authors found low to moderate correlation between salivary and plasma oxytocin, such as Grewen et al.[55] (r = 0.59) in mothers in response to infant contact and stress and Feldman et al.[56] (r = 0.41) in mothers and fathers interacting with their infants. Much further research is required to detail the mechanisms through which changes in brain oxytocin can be reflected in various peripheral systems.

**Table 1:** Comparison between peripheral (plasma and saliva) and central oxytocin concentrations (cerebrospinal fluid) (CSF: cerebrospinal fluid; EIA: enzyme immunoassay; RIA: radioimmunoassay)

Samples compared	Correlation	Species	Experimental setting	Assay used	Reference
Plasma and CSF	-0.149 (p = 0.352)	Human	Basal conditions	RIA	[52]
Plasma and CSF	0.00 (P = 0.99) (RIA) 0.48 (P = 0.02) (EIA) 0.80 (P < 0.01) (EIA) 0.59 (P = 0.04) (EIA)	Human and non-human primates	During surgery (human) and awake and anesthetized animals (non-human primates)	EIA (Enzo Life Sciences) RIA	[57]
Plasma, saliva and CSF	Saliva-CSF: 0.657 (P < 0.001) Saliva-Plasma: 0.361 (P = 0.010) Plasma-CSF: 0.417 (P = 0.003)	Human	Patients in neurocritical care	RIA	[51]
Saliva and plasma	-0.26 (P = 0.43)	Human	After intranasal oxytocin administration	EIA (Enzo Life Sciences)	[33]
Plasma and CSF	0.10 (P = 0.714)	Human	After intranasal oxytocin administration	RIA	[58]
Plasma and CSF	0.56 (P = 0.006)	Human	Anxiety	EIA (Enzo Life Sciences)	[59]
Saliva and plasma	0.59 (P = 0.022)	Human	Breats-feeding mothers	EIA (Assay Designs)	[55]
Saliva and plasma	0.252 (P = 0.180)	Human	Healthy young men	EIA (LUCIO- Medical ELISA Oxytocin)	[53]
Plasma and CSF	0.926 (P < 0.001)	Human	Headache	RIA	[60]
Plasma, saliva and CSF	Saliva-CSF: 0.624 (P < 0.001) Plasma-CSF: 0.407 (P < 0.001)	Human	Postpartum depression	EIA (Nanjing SenBeiJia Biologic Technology Co.)	[61]
Saliva and plasma	0.32 (P < 0.01)	Human	Depressive symptomatology	EIA (Assay Designs)	[62]
Saliva and plasma	0.41 (P < 0.001)	Human	Parents interaction with infants	EIA (Assay Designs)	[56]
Plasma and CSF	No significant	Human	Suicide attempters	RIA	[47]
Amygdala/Hippocampus and plasma	0.771 (P = 0.072) 0.600 (P = 0.208) -0.030 (P = 0.934) -0.029 (P = 0.957)	Rat and mice	After intranasal and peripheral oxytocin administration	RIA	[63]
Hypothalamic and serum	0.010 (P = 0.967) 0.596 (P = 0.006) 0.542 (P = 0.016)	Rat	Mother-pup interaction	EIA (Assay Designs)	[64]

Maybe one of the reasons for the divergences found in the relation between oxytocin in cerebrospinal fluid and other sample types, can be the different assays used. As an example, plasma and cerebrospinal oxytocin concentrations were not correlated (r = 0.00, P = 0.99) when radioimmunoassay (RIA) was used but showed a significant correlation (r = 0.80, P < 0.01) when an enzyme immunoassay (EIA) was used [57]. Also, these relations could be influenced by the concentration of oxytocin. For example, there is evidence for a positive association between central and peripheral oxytocin concentrations after intranasal oxytocin administration and after experimental stress induction; but this association did not appear under baseline conditions [65].

Initially, some authors did not recommend oxytocin measurement in saliva because its concentrations were very low and therefore, difficult to detect with the assays developed at that time [66]. The low values of oxytocin in saliva have been confirmed with studies reporting a range between 2 and 10 pg/mL in human saliva without extraction, that is more than 10-fold lower than the values in plasma (150–250 pg/mL) [3]. However, Carter et al. [3] used an EIA and concentrated samples, and accurately detected salivary oxytocin in humans, suggesting that measurements of biologically relevant changes in salivary oxytocin are possible. Some years later, other authors measured oxytocin in saliva with sensitive assays [55, 62] and demonstrated that oxytocin concentrations in saliva can change in stressful situations.

In animals, dogs and pigs have higher values of oxytocin in saliva than humans, with values usually higher than 200 pg/mL [43, 67]. However, different values can be obtained depending on the assay used. For example, in canine saliva a median value of 258 pg/mL (range = 207–471 pg/mL) was obtained with Arbor Assays kit (K048) and a median value of 679 pg/mL (range = 356–1073 pg/mL) with Cayman Chemical kit (Item #500,440) [67], while in case of an assay developed with AlphaLISA technology, the median value in dogs was 335 pg/mL ([68]. Also, oxytocin can be measured in saliva of goats (around 500 pg/mL) and cattle (around 200 pg/mL) [69]. In pig species, salivary oxytocin concentrations showed higher values in young individuals [70].

From the applicative point of view, changes in salivary oxytocin concentrations have been observed in both humans [71, 72] and animals [43, 67, 68] in different conditions. In human species, oxytocin can increase in saliva as after intranasal oxytocin administration; although part of this increase could result from a direct movement of mucus or it could be a residue of the spray administered present in the pharynx and oral cavity [73, 74]. In addition, it can increase in saliva in an affiliation relationship [56], lactating women [72], physiological relaxation and emotional excitation status [75], and after physical effort, psychological stress and sexual self-stimulation [71]. Oxytocin also has described to have an anti-stress function[76] showing higher values in saliva in patients with depressive symptoms [62]. In animals, oxytocin in saliva has been studied in human-animal interactions [69, 77], stroking in dogs[68, 78] and stress or situations of positive welfare [79, 80].

## Sample processing

It should be point out the importance of using standardized collection conditions and sampling devices. For example, in dogs, Sarstedt Salivette® yields oxytocin concentrations significantly lower than a SalivaBio Children’s Swab, and citric acid and eating immediately prior to the sample tended to increase oxytocin values [67].

Oxytocin measurements can be made on unprocessed samples or by performing a sample extraction. In addition, reduction/alkylation has been applied for sample processing. Also, a concentration can be done in cases that the limit of the assay detection is higher than the oxytocin levels of the sample to be measured [81]. Table 2 shows examples of the different sample treatment applied. Overall, there is a huge variation in the methods for sample treatment that researchers have used.

**Table 2:** Oxytocin values in different species in saliva (in case of various references, the values in the table indicate the lower and upper limits described in the references).

Species	Salivary concentration	Sample processing	References
Human	1.80–7.60 pg/ml	Extracted and concentrated	[55, 74, 82]
	1.5-51.05 pg/ml	Non-extracted and concentrated	[3, 35, 36, 71–73, 82]
	500–1200 pg/ml	Reduction-alkylation	[83]
Dog	27–418 pg/ml	Extracted	[77, 81]
	100–5000 pg/ml	Non-extracted	[68, 77, 78, 84]
	100–400 pg/ml	Reduction-alkylation	[68]
Cattle	0-3000 pg/ml	Non-extracted	[85]
	0-700 pg/ml	Reduction-alkykation	
	20–550 pg/ml	Extracted	[69]
Pig	129.6-814.2 pg/ml	Extracted	[69, 86]
	718.4–2140.0 pg/ml	Non-extracted	[79, 80]
	0-2500 pg/ml	Reduction-alkylation	[43]
Goat	100–1000 pg/ml	Non-extracted	[69]

### Sample extraction

The purpose of sample extraction is to obtain a higher purity of the analyte of interest from the sample prior to analysis with the elimination of potentially interfering molecules and the reduction of sample matrix effects [87]. In the particular case of oxytocin, the sample extraction removes the oxytocin bound to proteins and other molecules. Therefore, with this procedure the “free fraction” of oxytocin is measured. In general, lower values of oxytocin are obtained after this process, possibly due to the elimination of interfering molecules, that could artificially increase oxytocin values, and the matrix effect [87] and/or because extraction procedure eliminates oxytocin bound to other molecules, and only “free” oxytocin is measured [83].

There are different methods for sample oxytocin extraction. One of them is solid phase extraction (SPE) that uses different types of columns [67, 82, 88]. This procedure usually treats the sample with 0.1% trifluoroacetic acid (TFA-H_2_O), and after a centrifugation the supernatant is applied to the extraction column (equilibrated with acetonitrile and TFA- H_2_O). The eluate is evaporated, and the samples are reconstituted with assay buffer [57]. Alternatively, to SPE, a solvent extraction, using for example acetone, can be used [89]. The type of extraction can influence the final values, since the recovery of oxytocin values in plasma samples using SPE is higher than when using a solvent [82]. SPE columns are more efficient and accurate than acetone and are the preferred method for extracting oxytocin from serum or plasma samples [88].

There is controvery in the literature over whether oxytocin extraction is or not necessary. Some authors such as McCollough et al. [50], Leng and Sabatier[87] and Christensen et al.[90] recommended extraction, because non-extracted samples contain substances that are tagged as oxytocin in assays but are not oxytocin [87]. Also, it is described a high risk of matrix interference if samples are not extracted [90]. Considering these issues, analyses of unextracted plasma have been described as “no more than a random number generator” [87]. On the other hand, some studies report direct relationship between oxytocin concentrations determined in extracted and non-extracted samples, suggesting the no need of extraction. In rhesus monkeys, there was a high correlation (r = 0.89) between oxytocin levels in non-extracted and extracted serum samples [91]. Also, in some species such as dog, the high correlation (Arbor assays: r = 0.80, Cayman kit: r = 0.61) between the extracted and non-extracted saliva samples and the high spiking recoveries found in non-extracted saliva samples, make the extraction procedure not necessary [67]. Similarly, this has been recently described in saliva of pigs [86]. This could indicate that probably if the assay is adequate, sample extraction is not necessary, and this would avoid some risks and limitations associated to the extraction procedure such as:

- The lower oxytocin values that are obtained with extracted samples, could be due to the elimination of interfering substances but also due to the loss of the oxytocin of the sample during the procedure [66].
- The loss of correlation between analytical values ​​and biological effects that can be produced after the process of extraction [92].
- Analytical extraction is expensive and time consuming.

However, there are some available commercial kits that recommend the extraction of saliva samples for its measurement, like Arbor assay kit [93] or Cayman kit [69]. Despite to this, it is demonstrated the validity of measuring oxytocin using the Arbor Assays and the Cayman kit without extraction in plasma of mice (r = 0.95 between extracted and non-extracted samples with Arbor Assays) [94] and in saliva of dogs [67] (r = 0.80 in case of Arbor Assays and r = 0.61 in case of Cayman between extracted and non-extracted samples). However, these results are not so clear with other assays, such as in the case of Enzo kit, which shows less correlation than in case of Arbor Assays between extracted and non-extracted plasma sample of mice (r = 0.676), so there are differences between kits and it would be important to evaluate the need of sample extraction in each assay, as a part of its validation. before its use [94].

### Reduction/alkylation

Oxytocin in blood can bind to larger proteins, but also there is a fraction which is free. Brandtzaeg et al. [83] developed a reduction/alkylation procedure that broke the bonds between oxytocin and plasma proteins enabling the detection of total oxytocin, not only the oxytocin that is “free”. When they applied reduction/alkylation to plasma samples, obtained large increases in detectable oxytocin of human plasma compared with non-treated plasma. In addition, reduction/alkylation plasma samples yielded excellent linearity and parallelism when measured with commercial EIA kits. They indicated that total oxytocin may in many cases be better suited as a biomarker than the free fraction of oxytocin. This reduction and alkylation process could also affect to the molecular conformation of oxytocin by breaking the disulfide bridge and producing a linear form [39, 83].

López-Arjona et al.[43] developed two immunoassays with polyclonal and monoclonal antibodies for salivary oxytocin measurement. When they tested the assays after the reduction/alkylation treatment to saliva samples, the monoclonal antibody showed a high affinity by the total oxytocin (without significant differences before and after the treatment) and the polyclonal antibody showed a high affinity by bound oxytocin and other forms or metabolites of oxytocin. These results were obtained in saliva of porcine [43], canine[68] and bovine[85] species.

The performance of a reduction/alkylation procedure would be recommended at the initial stage of the analytical validation of any assay to determine which forms of oxytocin can recognize.

### Sample concentration

Due to the low oxytocin levels in human saliva, it has been described that for some methods, a sample concentration of at least 4-fold is necessary [3]. With this procedure, the oxytocin concentration in saliva would be in a reliable part of the standard curve of some commercial kits [72]. The most common procedure to do the sample concentration is by lyophilization. As an example, for doing a concentration of 4-fold by this method, a procedure that can be followed is to dry 1 ml of supernatant from each sample at 4 °C in a lyophilizer. Then the lyophilized material is reconstituted in 250 µL, resulting in a sample with a salivary concentration 4 times higher than the original [72]. Other authors chose to evaporate saliva samples and suspended with the assay buffer for oxytocin measurements [71].

In veterinary medicine, a previous concentration for the measurement of oxytocin in plasma has been recommended for some methods and species. For example, 10-fold in sheep, goat and pigs, 15-fold in horses and 20-fold in the cow has been recommended for the Enzo Life Sciences kit [81]. However, with other methods like Arbor Assays kit, Cayman kit or AlphaLISA technology it is not necessary to concentrate the dog saliva [77], pig saliva [43] and plasma samples of dog and cat [81].

### Storage

Although no reports in saliva have been found, it is described that the storage of samples in a range of temperatures from 0 to 37ºC until 18 h or several freeze/thaw cycles have minimal effects on oxytocin stability in plasma samples [82]. In urine samples, stability tests revealed that oxytocin concentrations degrade over time when stored at 4 °C but are little affected by repeated thawing [95].

## Assays for oxytocin measurement

The oxytocin sequence structure is highly conserved among mammals [96], so the different methods used for oxytocin measurement could recognized the oxytocin in all the species of mammals, however, it should be taken in consideration that different species have diverse oxytocin concentrations in biological fluids.

The main types of assays used for oxytocin measurements in saliva are:

### RIA

Although it was the method that was firstly used for the measurement of oxytocin [97, 98], it has some limitations such as:

- Low sensitivity in some cases that limits the detection of low oxytocin concentrations, existing various reports in which RIAs were not sensitive enough for detecting oxytocin in human plasma [47, 55, 89, 99], although some RIAs have very low detection limits such as 0.5 pg/sample for human saliva [71].
- Use of radioactive material and need of special conditions in the laboratory for its performance.

In some cases, the low sensitivity can be observed even after extraction and 10-fold concentration of human plasma samples, with 90% of human plasma samples showing values below the limit of detection of the RIA [82]. This has been also described in other species such as rat where Vecsernyés et al. [100] reported basal oxytocin for male rats to be 9.6 pg/ml, but the minimum detection limit for that assay was near 10 pg/ml.

### EIA

EIA assays have some advantages over available RIA kits, such as a longer shelf life of the reagents, the lack of radioactive materials and a wider detection range. Moreover, EIA requires sample volumes much smaller than those necessary for the RIA, although it depends primarily on whether an extraction is performed prior to assaying. The first EIA suitable for measuring oxytocin in plasma was developed by Prakash et al. [101], showing very close agreement with RIA method in the same samples after extraction.

In a study by MacLean et al. (2018), oxytocin concentrations in dog saliva using mass spectrometry (HPLC-MS) and EIA techniques were determined, demonstrating a high correlation (Arbor assays: r = 0.80, Cayman kit: r = 0.61) of oxytocin levels in the same samples analyzed with and without extraction. However, the oxytocin concentrations detected by EIA were much higher than those for mass spectrometry. This could be probably because the HPLC-MS detects the specific compound based on a known mass-to-charge ratio. By contrast, EIA may recognize not only the primary form of the target analyte, but also structurally related molecules, including precursor forms and biologically related metabolites [38].

Table 3 provides information about EIA kits that have been used for oxytocin measurements in saliva and other kind of samples. As it can be observed there is a variability in the detection limit of the different kits; with the EIA kit developed by Assay Designs, Inc. reporting a minimum detection limit of 4.68 pg/mL [92], whereas the kit of Cayman has a detection limit of 20 pg/mL. In general, it is a lack of knowledge about the form of oxytocin and possible oxytocin metabolites that the different assays detect.

**Table 3:** Commercial EIA kits used for oxytocin measurements in saliva (EIA: enzyme immunoassay)

Commercial kit	Method	Sensitivity	Species	Saliva processing	Other matrix samples used
Arbor assay	EIA	16.38 pg/ml	Dog [67] Western lowland gorilla [93]	Concentration	Urine
ALPCO	EIA	15 pg/ml	Rats [102] Human [62, 103]	Concentration	Plasma Cerebrospinal fluid Urine
Assay Designs	EIA	4.68 pg/ml	Human [3, 35, 36, 92]	Concentration	Plasma
Cayman Chemical	EIA	20 pg/ml	Dog [77, 78] Pig, cattle, goat [69]	Extracted and non-extracted	Plasma
Enzo Life Sciences	EIA	15 pg/ml	Dog, cat, cow, horse, pig, goat, sheep [81] Dog [104] Mice [105] Pig [106] Wild chimpanzees [107] Human [74, 108]	Lyophilized and 4-fold concentrated	Plasma Urine Cerebrospinal fluid

### HPLC-MS

HPLC is a technique that separates molecules based on their physical properties. It has been used for oxytocin quantification in saliva of dogs [67]. They found that 9 of 20 total samples were below the limit of detection of the assay (~ 2 pg/mL) and the remaining samples had a median concentration of 18 pg/ml (range = 8–49 pg/mL). This assay showed a positive correlation with extracted and non-extracted samples measured by EIA.

Recently, various researchers have used liquid chromatography coupled to tandem mass spectrometry to assess oxytocin levels in rat, human or mice [109, 110]. Also, others reported plasma oxytocin levels, following extraction, within the range of 1–10 pg/mL [83, 111]. However, Wang et al. [84] used a LC-MS method for oxytocin measurement in dog saliva with higher values ranged between 0 and 5000 pg/ml.

### AlphaLISA

AlphaLISA technology (PerkinElmer) is an amplified luminescent proximity homogenous assay that uses two bead types, donor and acceptor. The AlphaLISA assay is based on the principle that, when a laser beam is shone on a donor bead, a single oxygen molecule is generated, producing excitation at an adjacent acceptor bead and amplification of a fluorescent signal, which is detected and quantified. Some advantages of this type of immunoassay in comparison with conventional EIA are the small sample volume and requires fewer steps than EIA, with no need for washings. There are few studies that have measured oxytocin with AlphaLISA technology in saliva or other samples. In saliva of pig, canine and bovine species, AlphaLISA methods have shown a good linearity, precision and recovery [43, 68, 85, 86]. In addition, they can measure different oxytocin forms, such as free, bound, or other metabolites of oxytocin, depending on the antibody against oxytocin used in the immunoassay. Moreover, the AlphaLISA was sensitive for detecting changes in oxytocin measurements in different situations of stress or welfare in these species.

## Conclusions

Based in the current knowledge, it can be stated that (a) measurements in saliva samples can detect variations in oxytocin concentrations reflecting changes in physiological conditions and, in some cases, correlation between values of salivary oxytocin and cerebrospinal fluid concentrations have been found. (b) In cases of use of assays with a limit of detection not able to quantify oxytocin in saliva, the use of concentration procedures would be recommended, and controversy exists about the need of extraction, although last reports do not support this procedure. (c) There is a wide range of different assays for oxytocin measurements and in any case, it is important to use assays fully validated showing an adequate accuracy and precision to measure oxytocin in saliva in the species planned to be studied. In addition, it would be of interest to know which form of oxytocin (free, linked to proteins and/or oxytocin metabolites) the assay is measuring. Overall, there is a major need of more studies, knowledge, and assay standardization efforts in order to progress in the adequate and proper use of saliva for the measurement of oxytocin and also to determine all its possible applications.

## Data Availability

The data generated and analyzed during the current study are available from the corresponding authors on reasonable request.
